# ABO Blood System and COVID-19 Susceptibility: Anti-A and Anti-B Antibodies Are the Key Points

**DOI:** 10.3389/fmed.2022.882477

**Published:** 2022-04-25

**Authors:** Álvaro Tamayo-Velasco, María Jesús Peñarrubia-Ponce, Francisco Javier Álvarez, Ignacio de la Fuente, Sonia Pérez-González, David Andaluz-Ojeda

**Affiliations:** ^1^Haematology and Hemotherapy Service, University Clinical Hospital, Valladolid, Spain; ^2^BioCritic. Group for Biomedical Research in Critical Care Medicine, Valladolid, Spain; ^3^Centro de Investigación Biomédica en Red de Enfermedades Infecciosas (CIBERINFEC), Instituto de Salud Carlos III, Madrid, Spain; ^4^Pharmacological Big Data Laboratory, Pharmacology, Faculty of Medicine, University of Valladolid, Valladolid, Spain; ^5^Intensive Care Service, Hospital Universitario Sanchinarro, HM Hospitales, Madrid, Spain

**Keywords:** ABO blood group, COVID-19, anti-A antibody, SARS-CoV-2 spike protein, ACE2 (angiotensin converting enzyme 2)

## Abstract

The implication of the ABO blood group in COVID-19 disease was formulated early, at the beginning of the COVID-19 pandemic more than 2 years ago. It has now been established that the A blood group is associated with more susceptibility and severe symptoms of COVID-19, while the O blood group shows protection against viral infection. In this review, we summarize the underlying pathophysiology of ABO blood groups and COVID-19 to explain the molecular aspects behind the protective mechanism in the O blood group. A or B antigens are not associated with a different risk of severe acute respiratory syndrome coronavirus 2 (SARS-CoV-2) infection than that of other antigens. In this case, the cornerstone is natural anti-A and anti-B antibodies from the ABO system. They are capable of interfering with the S protein (SARS-CoV-2) and angiotensin-converting enzyme 2 (ACE2; host cell receptor), thereby conferring protection to patients with sufficient antibodies (O blood group). Indeed, the titers of natural antibodies and the IgG isotype (specific to the O blood group) may be determinants of susceptibility and severity. Moreover, older adults are associated with a higher risk of bad outcomes due to the lack of antibodies and the upregulation of ACE2 expression during senescence. A better understanding of the role of the molecular mechanism of ABO blood groups in COVID-19 facilitates better prognostic stratification of the disease. Furthermore, it could represent an opportunity for new therapeutic strategies.

## Introduction

At 2 years since the beginning of the COVID-19 pandemic (1, 2), people worldwide continue to suffer deaths and important changes in their lifestyles (3). Although vaccines are being encouraged to hinder the spread of this pandemic (4, 5), the pathophysiology of severe acute respiratory syndrome coronavirus 2 (SARS-CoV-2) is not well understood. Many studies identified multiple risk factors during the first wave to identify and treat susceptible patients early (6–8). Old age (9), male (10), and comorbidities, such as hypertension (11), were related to severity. Some routine biomarkers (12) and specific cytokines (13, 14) have also been proposed.

Similarly, a possible implication of the ABO blood group was formulated (15, 16). Currently, as multiple studies have reported (17–22), it seems clear that the A blood group is associated with more susceptibility and severe symptoms in COVID-19, while the O blood group shows protection against viral infection. Despite many descriptive studies on this tendency, few studies have focused on the implicated molecular mechanisms. Several studies have focused on angiotensin-converting enzyme 2 (ACE2) as the host cell receptor (23), S protein of the virus (24), and antigens or antibodies of the ABO system (25). However, no studies have specifically and directly deepened our understanding of the implications of ABO blood groups and their possible implications in developing future therapeutic strategies.

Therefore, we summarized the underlying pathophysiology of ABO blood groups and COVID-19. We exhaustively analyzed the role of A, B, AB, and O antigens in the disease and its molecular aspects. The functions of natural anti-A and anti-B antibodies are the cornerstone. We examined the importance of immunoglobulin (Ig) isotypes and their plasma concentrations by focusing on the consequences of immunosuppressive status according to the ABO system in patients with COVID-19. We examined how the complex interrelations between antibodies, the virus, and the host cell receptor relate to the protective molecular mechanism.

## Relationship Between ABO Blood Group and COVID-19 Severity and Susceptibility

### The Role of ABO Antigens in COVID-19

In 1901, Nobel Prize winner Karl Landsteiner discovered the ABO system (26). Erythrocytes, endothelial and epithelial respiratory cells, and digestive endothelial cells synthesize ABH carbohydrate epitopes. The addition of N-acetylgalactosamine or galactose to the H antigen (precursor chain) allows the appearance of A and B antigens, respectively. Thus, the O blood group only expresses the H antigen, whereas the AB blood group expresses both the A and B antigens (27, 28). In the Caucasian population, the O and A groups were the most frequent (45 and 40%, respectively), followed by the B group (11%) and AB group (4%). In contrast, group B is overexpressed in black and Asian populations (20 and 27%, respectively) (28). These differences in the ABO system are associated with some peculiarities. Blood group A is linked to hypercoagulability, cardiovascular events, and a higher risk of colon and gastric cancer (29). Group B is more susceptible to infections by *Escherichia coli* (30). The O blood group showed reduced thrombotic risk due to lower plasma von Willebrand factor (VWF) and coagulation factor VIII levels (29, 31).

Studies on COVID-19 also found more comorbidities in patients with the A blood group than those with the other groups (16, 17, 29). This subgroup of patients has a higher Charlson comorbidity index (32) and more cardiovascular diseases, especially hypertension (20), when infected with SARS-CoV-2. Moreover, according to the ABO blood group, these innate differences are not confounders. Multiple studies have confirmed the increased susceptibility, severity, and death risk in the A blood group, an independent risk factor for COVID-19 (17, 18, 20–22, 33). The implication of the ABO system was also strongly evidenced in a genome-wide associated study (GWAS) that identified a 3p 21.31 gene cluster related to the ABO blood group and respiratory failure in COVID-19 (34). We can expect new findings from genome-wide association analyses to explain better the importance of the ABO system in the severity and mortality of patients with COVID-19.

Descriptive and genetic studies based on ABO phenotypes found clear evidence about the implication of the ABO system in susceptibility and disease severity. However, no direct molecular interrelation between ABO system antigens and the virus has elucidated the mechanism involved in the susceptibility of the ABO blood group.

### Anti-A and Anti-B Antibodies Are the Cornerstones

Antigens of ABO blood group are present in the cell membrane. However, they do not directly modulate the SARS-CoV-2 infectious capacity. Natural anti-A or anti-B antibodies in patients with the A, B, or O blood groups are freely present in plasma, providing a decisive connection with the virus.

#### The Direct Connection Between Antibodies, S Protein of SARS-CoV-2, and ACE2 Receptors in the Host Cell

The infectious capacity of SARS-CoV-2 has been characterized previously. The virus binds to the cell surface *via* its S protein, cell receptor-binding domains (RBDs), and virus-cell membrane fusion domains (35). The S protein binds to the host cell receptor's ACE2 (36). ACE2 is present in virtually all organs, but lung alveolar epithelial cells and enterocytes of the small intestine (37) are important in this context. Moreover, the transmembrane protease serine subfamily member 2 (TMPRSS2), a cell surface protein expressed by endothelial cells in the respiratory and digestive tracts, is used by the virus for S protein priming (38). Enhanced entry correlated with optimal functions of both TMPRSS2 and ACE2. Similarly, in ACE2 expressing cells, dendritic-cell-specific ICAM3-grabbing nonintegrin (DC/L-SIGN) facilitates the infectious capacity; however, an adequate ACE2 correlation is required (39). These mechanisms promulgated in SARS-CoV-2 have also been confirmed in COVID-19 (40, 41). ACE2 is the main host cell receptor for the viral S protein (no other receptor has been discussed), and its function is probably improved by the proper interaction between TMPRSS2 and DC/L-SIGN (42).

**Figure 1:**
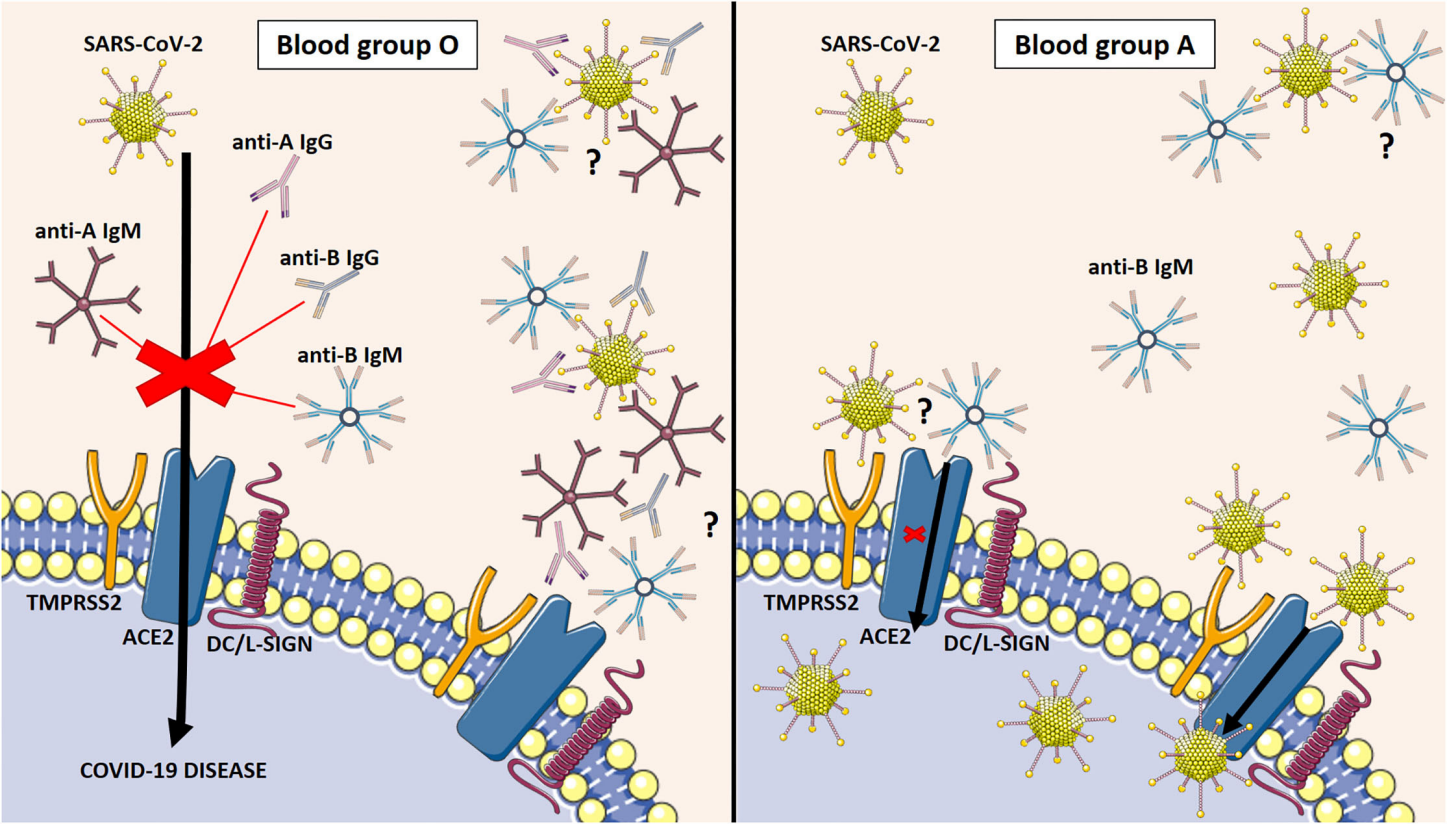
Molecular mechanism that explains the susceptibility and severity of COVID-19 disease depending on the ABO blood group. The presence of anti-A antibodies and probably anti-B antibodies inhibit the interaction between the S protein of the virus and the ACE2 on the cell surface in the O blood group (Left side). The absence of antibodies in the A blood group facilitates the entrance of SARS-CoV-2 into the host cell and the consequent viral infection (Right side). ACE2: angiotensin-converting enzyme 2. TMPRSS2: transmembrane protease serine subfamily member DC/L-SIGN; dendritic-cell-specific ICAM3-grabbing nonintegrin.; Natural antibodies bind glycosylated or carbohydrate epitopes in the S protein of SARS-CoV-2 (on top) or ACE2 (below).

Immunoglobulins can bind to or block different proteins. Anti-A and anti-B antibodies from the ABO system are natural Igs in serum. It has been reported that the presence of anti-A antibodies (and probably anti-B antibodies) prevents the interaction between the viral S protein of the virus and ACE2 on the cell surface (Figure 1). The molecular mechanism is not yet fully understood. However, several hypotheses have been proposed. It seems that carbohydrates or glycosylated epitopes are present in the cell membrane of both SARS-CoV-2 and ACE2 and it is known the strong binding between natural antibodies from the ABO system and carbohydrate molecules, such as A or B antigens. On the one hand, the S protein could be decorated with A or B carbohydrate epitopes able to be recognized by the natural anti-A or -B antibodies from blood group O, B, and A individuals (24). On the other hand, natural anti-A or -B antibodies can directly bind to the ACE2 glycosylated region (43). In this case, possible competitive inhibition of ACE2 by both natural (anti-A/anti-B) antibodies and SARS-CoV-2 may induce early ACE2 downregulation in blood group O, increasing the production of multiple inflammatory cytokines (44) in the first step of the infection. Patients with the O blood group suffered a consequent cytokine drop during the hospital stay, while non-O patients maintained their cytokine levels associated with hyperinflammation. An early, effective, and moderate cytokine release functions in immunocompetent patients, while disease severity is linked to persistent immune dysregulation after infection, associated with high cytokine levels for days or weeks. These findings could explain the optimal activation of the immune response and the effective viral clearance of SARS-CoV-2 infection in the patients with the O blood group. In any case, the interaction between natural antibodies and ACE2 or S proteins should prevent viral infection *via* transfusion rules. Therefore, natural anti-A or -B antibodies protect patients with the O blood group against severe disease and mortality in COVID-19. Comparatively, antibodies operate *via* the same mechanism as future specific treatments against SARS-CoV-2 (45). The more anti-A or-B antibodies present in the plasma (O blood group), the reduced infectious capacity (19). In contrast, the absence of antibodies (AB blood group) or one of them (A or B blood groups) is associated with a higher risk for poor outcomes in COVID-19 (20). Descriptive and epidemiological studies have corroborated this tendency during the pandemic in all populations (15, 17, 18, 20–22, 32, 33, 46).

#### Immunoglobulin Isotype of Anti-A and Anti-B Antibodies

**Figure 2:**
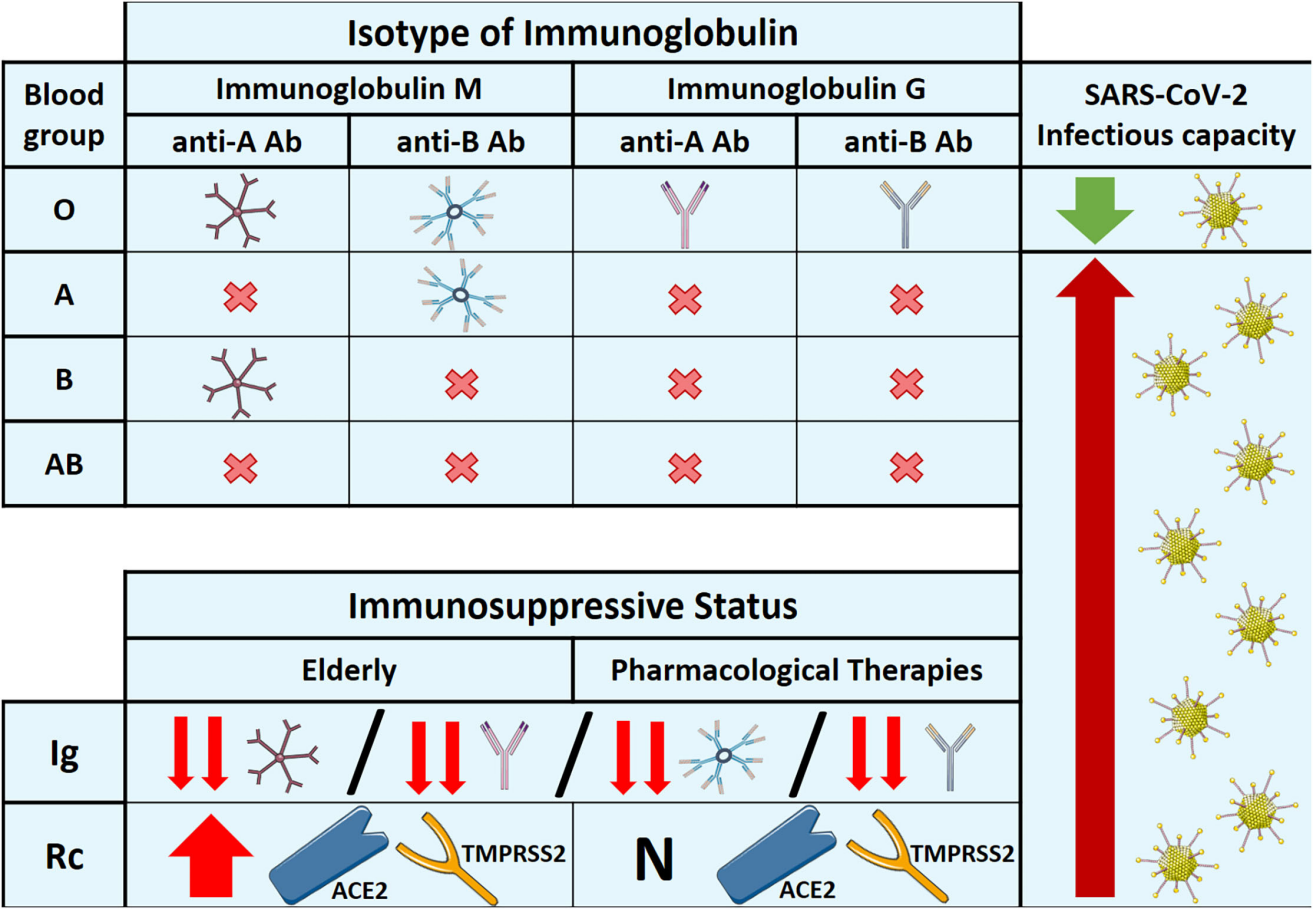
Specific isotype of immunoglobulin in each ABO blood group, expression of natural antibodies and receptors in immunosuppressive situations, and their relationship with the infectious capacity of SARS-CoV-2. Ab: antibody. Rc: receptor. Ig: immunoglobulin. ACE2: angiotensin-converting enzyme 2. TMPRSS2: transmembrane protease serine subfamily member. N; normal expression.

Natural anti-A or -B antibodies from the ABO system differ from most naturally occurring antibodies because of their exclusive expression in individuals lacking the corresponding antigen (A or B antigen)(47). They exhibit high polyspecificity and polyreactivity to multiple antigens, not only those included in the ABO system (48). The main isotype of natural Ig is M (IgM) in all ABO blood groups with specific natural antibodies (A, B, and O blood groups), reaching all groups with similar plasma concentrations of IgM antibodies. By cons, the presence of anti-A/B antibodies with the IgG isotype was restricted to the O blood group (Figure 2). Indeed, anti-A or anti-B IgG were found in almost 90% (34/38) of O blood group donors (predominance of IgG2). Meanwhile, only 14% of patients with the A blood group had anti-B IgG, and 4% with B blood group had anti-A IgG. None of the AB blood group samples contained anti-A or anti-B antibodies of any isotype (IgM or IgG) (25). Until now, there has been no explanation for this finding. It would be relevant, for example, in hemolytic disease of the newborn because only newborns of blood group O mothers develop the hemolytic disease after ABO-incompatible pregnancies (49). In COVID-19, studies have shown that patients with the O blood group are under-represented, whereas patients with groups A, B, and AB are over-represented (20). We previously explained that the higher the plasma concentration of natural anti-A or anti-B antibodies (O blood group), the higher the protective effect against SARS-CoV-2 infection. Nevertheless, it is not only the plasma level of natural antibodies but also the isotype of Ig. IgG (restricted to the O blood group) may strongly avoid the interaction between ACE2 and the S protein compared to the IgM isotype.

#### Immunosuppressive Status and Plasma Antibody Levels

A strong immune system is crucial for overcoming infections. It includes both an optimal innate and adaptive immune response, with adequate antibody production by B cells. Unfortunately, many situations can weaken the immune system, reducing cell-mediated immune function and humoral immune responses. This decline in immune capacity is associated with reduced antibody levels, making individuals more suitable for infections and disease severity. Older individuals are one of the most recognized cases (9). Aging reduces the production of B and T cells in the bone marrow and thymus and diminishes the function of mature lymphocytes in secondary lymphoid tissues (50). Similarly, immunosuppressive treatments (glucocorticoids, cytotoxic drugs, other immunomodulatory agents, or new immunosuppressive therapies) can also compromise the immune system (51). In addition, infections can have immunosuppressive effects in the local environment (52), increasing susceptibility and severity of infectious diseases and decreasing the efficacy of vaccination (53). Moreover, different studies have revealed, in severe cases of COVID-19, that the presence of immune downregulation with profound immunosuppression was the primary phenomenon. Immunological alterations vary and are classified into different subsets or phenotypes. One of these immunophenotypes is characterized by the coexisting alterations in T cells' numbers, subset composition, cycling, activation, and gene expression (54, 55).

It has been shown for SARS-CoV that the interference between natural anti-A antibodies in the O blood group was dose-dependent and still detected at a plasma dilution of up to 1/32. Indeed, patients with the O blood group with low anti-A antibodies were not inhibitory in the host cell adhesion assay (24). The lack of or drop in antibodies due to any immunosuppressive situation creates ABO discrepancies (56). Therefore, it is of value to determine whether the ABO group performed both forward (red blood cell antigen) and reverse (anti-A and anti-B antibodies in plasma). Once we know the importance of natural antibodies from the ABO system in COVID-19, we should evaluate only the reverse type in terms of protection against viral infection. Accordingly, patients with the O, A, or B blood groups (forward type) that associate lack of antibodies would behave as the AB blood group (reverse type). The current situation favors infections and worsens outcomes for a large number of people, especially older adults and patients who are immunosuppressed.

While aging decreases plasma levels of antibodies, studies have demonstrated increased ACE2 and TMPRSS2 expression in older adults (10, 57). The first study described a significant expression of ACE2 in older males in both mouse models and human organs (10). The second study demonstrated the overexpression of ACE2 and TMPRSS2 in the upper respiratory tract of aged ferrets compared to young animals (57). Moreover, a recent study found that ACE2 levels increase during aging in mouse and human lungs due to telomere shortening or dysfunction (58). It involves the transcriptional level, where ACE2 promoter activity is dependent on DNA damage response (58). Therefore, both the upregulation of ACE2 and the decrease in antibodies make the elderly more susceptible to severe infection by SARS-CoV-2 (Figure 2).

## Discussion

After this exhaustive assessment regarding the implications of the ABO blood system in COVID-19, we make the following key points: i) The presence or absence of any antigen of the ABO system is related to different susceptibilities, presenting more comorbidity in patients with antigen A (A blood group), while the absence of antigens (O blood group) is associated with lower thrombotic and cardiovascular risk. This is one of the reasons why the number of patients infected with SARS-CoV-2 who were hospitalized with worse outcomes belongs to the non-O blood group. However, there is no direct molecular relationship between ABO system antigens and the virus that can explain the true mechanism involved in the susceptibility of the ABO blood group. ii) Natural anti-A and -B antibodies from the ABO system are capable of interfering with the S protein (SARS-CoV-2) and ACE2 (host cell receptor). The presence of high plasma concentrations of antibodies in the O blood group confers greater protection to these patients. iii) The isotype of natural antibodies would be decisive because the A, B, and O blood groups present IgM, but only the O blood group presents anti-A and anti-B IgG antibodies in the plasma. iv) Immunosuppressive status, such as in older adults and patients with some diseases or undergoing pharmacological treatments, is associated with a lack of antibodies. This creates the ABO discrepancies. Patients with the O, A, or B blood groups would behave as patients with the AB blood group, making them more susceptible to infection.

Some questions might be interesting to consider and could open future investigations in this area. The first is related to the exact mechanism by which natural antibodies from the ABO blood system avoid the interaction between the S protein of the virus and ACE2 on the cell surface (20, 24, 43, 44). An experimental model is required to understand whether antibodies only block the S protein, perhaps together with SARS-CoV-2, to competitively inhibit ACE2 in host cells or whether both molecular mechanisms are possible. These findings would help us to underline the pathophysiology of the ABO blood groups in the same way that the lower plasma von Willebrand factor (VWF) and coagulation factor VIII levels in the O blood group are well described (29, 31). For example, suppose our natural anti-A or -B antibodies would constantly bind or block ACE2. In that case, O blood group individuals could present persistent ACE2 downregulation, resulting in increased production of multiple inflammatory cytokines (44). Furthermore, these antibodies might be associated with protection against cardiovascular diseases, similar to ACE inhibitors, conferring a lower risk in the O blood group. Therefore, it would be necessary for anti-A and anti-B antibodies to bind to the same proteins, or one of them must demonstrate more affinity or interfere with SARS-CoV-2 more efficiently.

Another important issue is the antibody isotype. As mentioned before, the IgM isotype is present in A, B, and O blood groups, while anti-A and anti-B with IgG isotypes are almost unique to the O blood group (25). The better outcome of the O blood group is confirmed in COVID-19, but this effect depends on the higher plasma level of natural antibodies compared to the rest of the blood groups (24), or perhaps IgG isotypes confer more protection or both. The IgG isotype interferes more strongly than IgM, explaining the protective status of the O blood group. However, elucidation would require complicated and specific laboratory assays, COVID-19 cases, healthy donors, and all blood groups and isotypes of Igs.

Finally, studying older patients might determine whether the upregulation of ACE2 or the decrease in antibodies with senescence is significant (10, 57). Moreover, the implications of ACE2 upregulation would lead to specific studies based on different symptoms in old and young patients. Patients with ACE2 overexpression in the gastrointestinal tract are associated with more diarrhea (59). In fact, there is evidence demonstrating a direct association between endothelitis and severe COVID-19 (60). Therefore, ACE2 may be a relevant factor in this phenomenon.

## Conclusion

In conclusion, natural anti-A and B antibodies from the ABO system interfere with the S protein (SARS-CoV-2) and ACE2 (host cell receptor), conferring protection to patients with sufficient antibodies (O blood group). The titers of natural antibodies and IgG isotype (specific to the O blood group) are determinants of susceptibility and severity. Older adults are associated with a higher bad outcomes risk due to the lack of antibodies and the upregulation of ACE2 expression. There is no doubt that more investigations would be beneficial to understand the role and molecular mechanism of ABO blood groups in COVID-19 fully and help develop novel therapeutic strategies.

## Author Contributions

ÁT-V, MP-P, and FÁ conceptualized the study. ÁT-V, IF, SP-G, and DA-O contributed to methodology and investigation. ÁT-V wrote the manuscript. ÁT-V and DA-O contributed to figures. MP-P, FÁ, and DA-O wrote and reviewed the article. ÁT-V and DA-O visualized the study. All authors critically revised the manuscript, reviewed the final version, and agreed with the content of the work.

## Funding

This research was funded by the Instituto de Salud Carlos III (CB21/13/00051 and COV20/00491) and Junta de Castilla y Leon (18IGOF and GRS COVID 108/A/20).

## Conflict of Interest

The authors declare that the research was conducted in the absence of any commercial or financial relationships that could be construed as a potential conflict of interest.

## Publisher's Note

All claims expressed in this article are solely those of the authors and do not necessarily represent those of their affiliated organizations, or those of the publisher, the editors and the reviewers. Any product that may be evaluated in this article, or claim that may be made by its manufacturer, is not guaranteed or endorsed by the publisher.
